# Interaction between polymorphisms of the Human Leukocyte Antigen and HPV-16 Variants on the risk of invasive cervical cancer

**DOI:** 10.1186/1471-2407-8-246

**Published:** 2008-08-22

**Authors:** Patricia S de Araujo Souza, Paulo C Maciag, Karina B Ribeiro, Maria Luiza Petzl-Erler, Eduardo L Franco, Luisa L Villa

**Affiliations:** 1 Ludwig Institute for Cancer Research, São Paulo, Brazil; 2 Department of Biochemistry, University of São Paulo, São Paulo, Brazil; 3 Hospital do Cancer A C Camargo/Fundação Antonio Prudente, São Paulo, Brazil; 4 Department of Genetics, Universidade Federal do Paraná, Curitiba, Brazil; 5 Departments of Oncology and Epidemiology, McGill University, Montreal, Canada; 6 Division of Cellular Biology, Brazilian National Cancer Institute, Rio de Janeiro, Brazil; 7 Advaxis Inc, North Brunswick, USA

## Abstract

**Background:**

Persistent infection with oncogenic types of human papillomavirus (HPV) is the major risk factor for invasive cervical cancer (ICC), and non-European variants of HPV-16 are associated with an increased risk of persistence and ICC. HLA class II polymorphisms are also associated with genetic susceptibility to ICC. Our aim is to verify if these associations are influenced by HPV-16 variability.

**Methods:**

We characterized HPV-16 variants by PCR in 107 ICC cases, which were typed for *HLA-DQA1*, *DRB1 *and *DQB1 *genes and compared to 257 controls. We measured the magnitude of associations by logistic regression analysis.

**Results:**

European (E), Asian-American (AA) and African (Af) variants were identified. Here we show that inverse association between DQB1*05 (adjusted odds ratio [OR] = 0.66; 95% confidence interval [CI]: 0.39–1.12]) and HPV-16 positive ICC in our previous report was mostly attributable to AA variant carriers (OR = 0.27; 95%CI: 0.10–0.75). We observed similar proportions of HLA DRB1*1302 carriers in E-P positive cases and controls, but interestingly, this allele was not found in AA cases (p = 0.03, Fisher exact test). A positive association with DRB1*15 was observed in both groups of women harboring either E (OR = 2.99; 95% CI: 1.13–7.86) or AA variants (OR = 2.34; 95% CI: 1.00–5.46). There was an inverse association between DRB1*04 and ICC among women with HPV-16 carrying the 350T [83L] single nucleotide polymorphism in the *E6 *gene (OR = 0.27; 95% CI: 0.08–0.96). An inverse association between DQB1*05 and cases carrying 350G (83V) variants was also found (OR = 0.37; 95% CI: 0.15–0.89).

**Conclusion:**

Our results suggest that the association between HLA polymorphism and risk of ICC might be influenced by the distribution of HPV-16 variants.

## Background

Invasive cervical cancer (ICC) is one of the leading causes of cancer-related death in women in developing countries. According to the WHO, the age-adjusted incidence rate of ICC in Brazil is 23.4 per 100,000 women [1], making it the second most common cancer in Brazilian women. The major risk factor is persistent infection with oncogenic types of human papillomavirus (HPV) with the contribution of additional co-factors such as smoking and oral contraceptive use. A strong association exists between persistent HPV infections and risk of squamous intraepithelial lesions (SIL), particularly for HPV types 16 and 18 [2]. HPV DNA sequences are found in 2% to 44% of sexually-active asymptomatic women [3], but virtually all cervical carcinomas contain DNA of the high-risk types [4]. However, HPV infection is necessary but not sufficient to cause the development of ICC.

HPV-16 is the most common type found in ICC and in healthy women. Investigations of HPV-16 sequence variability worldwide suggest that the virus evolved along five major phylogenetic branches that largely reflect the ethnicity of the human host populations [5]. Studies from different populations described that non European variants, mainly from the Asian-American branch, are associated with higher risks of HPV persistence and CIN development [6], as well as ICC [7]. Different biological and biochemical properties have already been attributed to naturally occurring variants of HPV-16 [8-11], and a differential risk for HPV persistence and ICC was also associated with some HPV-16 variants [12,13].

The increased rate of HPV related diseases in patients with cellular immunodeficiency suggests an important role of the immune response in the control of HPV infection [14]. Due to the crucial function of HLA class II molecules on antigen presentation to CD4 T cells, as well as the high polymorphism of HLA genes, many studies investigated associations between HLA class II alleles and HPV associated diseases [[15-17], for review see [18]].

A decreased risk of ICC was observed in carriers of DQB1*05 in our previous case-control study conducted in a Northeastern Brazilian population [19], and similar associations were found in studies conducted in the British population [20,21]. Positive associations with HLA-DRB1*15-DQB1*0602 were reported in Brazilian [19], British [20] and Swedish women [22], as well as Hispanics from New Mexico [23]. However, this haplotype was inversely associated with HPV-16 high-grade SIL (HSIL) in a study performed in United States [24]. The conflicting data concerning some of HLA associations among different populations may be influenced by HPV-16 variability, because variants can be immunologically distinct, since some sequence changes occur in potential HLA class II and I epitopes.

Previous studies were conducted to investigate the association between HPV-16 variants and HLA class II polymorphism in different populations. In Japanese women, DRB1*1501 and DQB1*0602 alleles are associated with ICC positive for HPV-16 prototype [25]. A study done with Swedish, Italian and Czech women revealed a trend for a positive association between carriers of DRB1*04-DQB1*03 haplotypes and ICC positive for E6 83V variants [26]. The same amino acid substitution was associated with HLA-DRB1*07 [27] and *DR4*-*DQ3 *[28] in Dutch and Swedish populations, respectively. Conversely, no associations were found in a British study [20].

To investigate if the association pattern between HLA class II genes and ICC is dependent on the distribution of HPV-16 variants, we evaluated the HPV-16 variability in 107 patients enrolled in a case-control study [19], previously analyzed for *HLA-DRB1*, *DQB1 *and *DQA1 *polymorphisms.

## Methods

### Samples

The 107 HPV-16 positive cases and 257 controls included in the present study were selected from an epidemiological study of ICC and HPV infection previously conducted in Brazil, whose details have been presented elsewhere [29,19]. Cases were women with histopathological confirmation of squamous ICC admitted for diagnosis and treatment at the Napoleão Laureano Hospital, in João Pessoa, Brazil, between 1986 and 1990. Controls were women with normal or inflammatory Pap smears selected from a citywide opportunistic screening program carried out at the same hospital. Written informed consent was obtained from all patients and controls, and ethical review committee of the Hospital Napoleão Laureano and of the Hospital do Cancer A C Camargo/Fundação Antonio Prudente approved the study. Risk factor information was obtained through a standardized interview carried out by a trained nurse using a structured questionnaire. Ethnicity was identified by the nurse according to previously designated categories (white, mulatto and black). A cervical cell specimen was collected from each control using a cytobrush, and tumor biopsies were obtained from all cases. Clinical specimens were prepared and submitted to cytological or histological examination, and the remaining cells and tissues were stored at -20°C and shipped in dry ice to the Ludwig Institute for Cancer Research (São Paulo, Brazil) to DNA extraction. Aliquots of the purified DNA were used for HPV detection and typing, characterization of HPV-16 variants as well as for HLA typing.

### HPV typing

DNA extraction and HPV detection and typing were performed by standard techniques [29]. Briefly, DNA samples from cases and controls were submitted to PCR-based amplification of a 450 bp segment in the L1 viral gene with MY09 and MY11 primers [30]. PCR products were dot blotted in a Nylon membrane and hybridized with individual ^32^P-labeled oligonucleotide probes specific for HPV types 6, 11, 16, 18, 26, 31, 33, 35, 39, 40, 42, 45, 51–59, 66, 68, 70, 71, 72, 81, 82, 83 and 89 [31,32].

### Characterization of HPV-16 variants

HPV-16 variants were characterized for *E6 *and *L1 *genes by nested PCR, followed by hybridization with oligonucleotide probes [33]. The probes sequences and washing temperatures were as described by before [33], except the *E6 *350G and 403A, washed at 47°C. The hybridization pattern of each sample with all the probes allows the classification of variants into European (E), Asian-American (AA), African-1 (Af-1) and African-2 (Af-2) branches, and two additional subclasses within E and AA branches [34]. Variant designation was done according to previous report [35].

Some specimens were further characterized by sequencing analysis of viral gene fragments. L1 PCR products were cloned using the Sure Clone ligation kit (Amersham Pharmacia, New Jersey, USA) and transformed into *E. coli*, strain DH5α. E6 PCR products cloning were done with TOPO TA Cloning kit (Invitrogen, Carlsbad, USA) and transformed into TOP10 Chemically competent *E.coli *(Invitrogen, Carlsbad, USA). Clones were screened by PCR with plasmid specific primers, and these PCR products were sequenced using Big-Dye Terminator (Applied Biosystems, Foster City, USA) and one of the primers used in the PCR. Sequences were analyzed in an ABI3100 sequencer (Applied Biosystems, Foster City, USA). To identify infections by multiple variants, five clones from each transformation were selected for sequencing analysis. Samples were classified as positive for multiple infections only if clones from different HPV-16 branches were identified in one gene fragment and confirmed in the other (*E6 *and *L1*).

### HLA typing

All the 257 controls and the 112 HPV-16 positive cases used in this study had been previously typed for HLA class II genes [19]. Typing was done by PCR-amplification of the 2^nd ^exon of *HLA-DQA1*, *DQB1 *and *DRB1 *genes, followed by hybridization with sequence-specific oligonucleotide probes [36].

### Statistical analysis

To evaluate the differences in the distribution of HLA group frequencies between cases carrying each HPV-16 variant and controls, odds ratios (OR) and respective 95% confidence intervals (95% CI) were calculated. Crude and age- and ethnicity-adjusted ORs were computed by unconditional logistic regression, using SPSS statistical software (version 11.0). In our previous study [19] other variables (level of education, income, consumption of alcoholic beverages other than beer or sugar cane distillates) were also controlled for because of their association with ethnicity. In the interest of precision, however, as similar estimates were obtained using models based on both sets of confounders we used only adjustment for age and ethnicity. All the associations that were analyzed are reported.

## Results

HPV-16 variants from E, AA, Af-1 and Af-2 branches were identified in 107 cases out of 112 HPV-16 positive ICC cases, because five samples were not included in variant analysis due to PCR failure or disagreement between variant identity in E6 and L1 genes. Although we detected 36 HPV positive samples in the control group [19], only 12 were HPV-16 positive. Due to this small number of carriers, we did not characterize HPV-16 variability in the control group. The frequency of cases harboring AA variants was similar to the frequency of cases with E variants, while a low proportion of African variants was observed, being 8 of Af-2 branch, and only one Af-1 variant (Table 1). We identified double infections by variants from different branches in 15 cases, 13 of which were carriers of AA and European prototype (E-P) variants, one positive for E-P and Af-2, and the other one carrying AA and Af-2 variants. Only one case harbored two different HPV-16 variants of the same branch, the European. The AAa variant, which belongs to the AA branch, was the most frequent HPV-16 variant in this series. From the same branch, we identified one NA-1 variant. The second most common variant was the E-P, from the E branch. We also identified an E-6994A, an E-G131T and 4 E-350G variants.

**Table 1:** Distribution of HPV-16 variants in cervical biopsies from HPV-16 positive ICC cases

Branch	Variants	E6 substitutions^a^				All HPV-16 cases	HPV-16 exclusively^d^
				
		10	14	78	83	Carriers (%)^b ^n = 107^c^	Carriers (%) n = 69
E						54 (50.5)	28 (40.6)
	E-P	A	Q	H	L	*49 (45.8)*	*23 (33.3)*
	*E-350G*	-	-	-	V	*4*	*3*
	*E-6994 A*	-	-	-	-	*1*	*1*
	*E-G131T*	G	-	-	-	*1*	*1*
AA						59 (55.1)	
	AAa	-	H	Y	V	*58 (54.2)*	*37 (53.6)*
	NA-1	-	H	Y	V	*1*	*0*
Af-1	Af-1	T	D	Y	-	1	0
Af-2	Af-2	I	D	Y	-	8 (7.5)	4 (5.7)

a) -: Residue identical to the E-P;

b) Percentages are shown only for variant branches and for the most common variant groups;

c) The total number of HPV-16 variants exceeds 107 because we included cases with infection by more than one variant;

d) Co-infections with other types excluded.

In our previous case-control study performed with these samples, we have reported that DRB1*15 and the DRB1*1503-DQB1*0602 haplotype were positively associated with ICC. On the other hand, DRB1*0101 and DQB1*05 were inversely associated with this disease [19]. As HPV-16 was the most common type found in those samples, we extended the type-specific analysis and the most relevant results are shown in Table 2.

**Table 2:** ORs and 95% CIs for HPV-16 positive ICC according to HLA.

	Controls (n = 257)	HPV-16 Cases (n = 112)			
		
HLA			Crude	Adjusted^d^	
				
	C/N^a^	C/N	OR^b^	OR	CI^c^
*DQA1*					
*0102	80/177	39/73	1.18	1.22	0.73–2.04
*DQB1*					
*0501	71/186	25/87	0.75	0.72	0.40–1.27
*06	87/170	46/66	1.36	1.32	0.80–2.17
*0602	44/213	30/82	1.77	1.59	0.89–2.83
*DRB1*					
*01	52/205	22/90	0.96	0.89	0.49–1.64
*0102	29/228	12/100	0.94	0.83	0.38–1.79
*04	74/183	23/89	0.64	0.67	0.38–1.19
*13	61/196	19/93	0.66	0.78	0.42–1.45
*1302	27/230	4/108	0.32	0.43	0.14–1.32
*DRB1-DQB1 haplotypes*					
0102–0501	29/228	12/100	0.94	0.59	0.23–1.51
15-0602	33/224	28/84	2.26	2.12	1.15–3.93
1503-0602	14/243	15/97	2.68	2.77	1.20–6.39
08041-0301	5/252	3/109	1.39	0.88	0.14–5.47
09012-0201/02	3/254	3/109	2.33	3.02	0.56–16.29

a) C/N: Carriers/Non carriers;

b) OR: Odds Ratio;

c) CI: Confidence Interval;

d) Adjusted for age and ethnic group.

To perform the analysis of HLA distribution we first excluded 33 cases that contained HPV types other than 16, and then excluded 5 cases that were positive for more than one HPV-16 variant, as the influence of one variant in immune response to another is not clear. We therefore evaluated the HLA distribution in 69 cases, which were positive for HPV-16 only and had a single variant infection, and 257 controls. HPV-16 variants distribution in these cases is also shown in Table 1.

The comparison of *HLA-DQA1*, *DQB1 *and *DRB1 *alleles' distribution between controls and cases, which were stratified according to major variants E-P and AA found in this sample, is presented on Table 3. We observed that the proportion of HLA-DQA1*0101/04 carriers was lower in cases positive for AA variants than in controls (Table 3). Due to linkage disequilibrium, this allele is commonly found in haplotypes with DQB1*05 and DRB1*01 alleles. As expected, an inverse association between HLA-DQB1*05 and DQB1*0501 carriers was also observed with AA variant cases (Table 3). Interestingly, the allele DRB1*1302 was not found among AA positive cases (p = 0.03, Fisher exact test), although its frequency was not statistically different between controls and ICC cases positive for E variants (Table 3).

**Table 3:** ORs and 95% CIs for ICC HPV-16EP or AA positive according to HLA.

	Controls	AA Cases			EP Cases		
			
HLA	C/N	C/N	Crude	Adjusted	C/N	Crude	Adjusted
							
	Controls	Cases	OR	OR (95% CI)	Cases	OR	OR (95% CI)
	n = 257	n = 37			n = 24		
DQA1							
*0101/04	79/178	5/32	0.35	0.34(0.12–0.93)	9/15	1.35	1.31 (0.53–3.25)
*0102	80/177	10/27	0.82	0.82(0.37–1.83)	12/12	2.21	2.32 (0.96–5.62)
DQB1							
*0301	78/179	9/28	0.74	0.74(0.32–1.70)	8/16	1.15	1.08 (0.42–2.75)
*05	95/162	5/32	0.27	0.27(0.10–0.75)	10/14	1.22	1.29 (0.53–3.14)
*0501	71/186	4/33	0.32	0.30 (0.10–0.92)	9/15	1.57	1.56 (0.63–3.89)
*06	87/170	16/21	1.49	1.49 (0.72–3.08)	12/12	1.95	2.02 (0.84–4.88)
*0602	44/213	9/28	1.56	1.45 (0.62–3.39)	8/16	2.42	2.13 (0.83–5.50)
DRB1							
*01	52/205	4/33	0.48	0.45 (0.15–1.37)	7/17	1.62	1.51 (0.57–4.03)
*0102	29/228	1/36	0.22	0.18 (0.02–1.44)	6/18	2.62	2.18 (0.75–6.29)
*04	74/183	10/27	0.92	0.94 (0.42–2.09)	3/21	0.35	0.34 (0.10–1.21)
*13	61/196	9/28	1.03	1.21 (0.52–2.78)	5/19	0.85	1.09 (0.37–3.16)
*1302	27/230	0/37	0.00		3/21	1.22	1.62 (0.42–6.16)
*15	34/223	10/27	2.43	2.34 (1.00–5.46)	8/16	3.28	2.99 (1.13–7.86)
*1503	14/243	5/32	2.71	2.62 (0.83–8.27)	4/20	3.47	3.75 (0.99–12.42)
*DRB1-DQB1 haplotypes*							
0102-0501	29/228	1/36	0.22	0.18 (0.02–1.44)	6/18	2.62	2.18 (0.75–6.29)
15-0602	33/224	8/29	1.87	1.84 (0.75–4.53)	8/16	3.39	3.08 (1.17–8.14)
1503-0602	14/243	5/32	2.71	2.62 (0.83–8.27)	4/20	3.47	3.50 (0.99–12.42)
08041-0301	5/252	0/27			2/22	4.58	3.59 (0.48–27.13)
09012-0201/02	3/254	1/36	2.35	2.62 (0.25–27.79)	1/23	3.68	5.00 (0.47–53.36)

A higher frequency of HLA-DQA1*0102 was found in cases positive for E variants (adjusted OR = 2.32, 95% CI: 0.96–5.62; Table 3; p = 0.06) than in controls, but not in those associated with AA variants (Table 3). Although the DQA1*0102 allele can be found in different *DRB1-DQB1 *haplotypes, we found it mainly with DRB1*15 and DQB1*0602 alleles. The association with DRB1*15 was comparable between European variants (OR = 2.99; 95% CI: 1.13–7.86; p = 0.03) and AA variants (OR = 2.34; 95% CI: 1.00–5.46, p = 0.05) (Table 3). In both European and AA groups, the DRB1*1503 allele had the highest OR values (adjusted OR = 3.75; 95% CI: 0.99–12.42; p = 0.05 and OR = 2.62; 95% CI: 0.83–8.27; p = 0.10, respectively). DRB1*15 alleles are found in linkage disequilibrium with DQB1*0602. However, it is worth noting that this allele was found, in most cases, in linkage with DRB1*15, whereas in the controls, a larger proportion of DQB1*0602 was found in linkage with DRB1*11 alleles.

The only statistically significant result in the comparison of *HLA-DRB1-DQB1 *haplotypes in the stratified analysis was an association between DRB1*15-DQB1*0602 and E-variant cases (p = 0.02, Table 3). Similar to the trends observed for the distribution of alleles, we found a lower frequency of the DRB1*0102-DQB1*0501 haplotype in patients carrying AA variants (adjusted OR = 0.18; 95% CI: 0.02–1.44; Table 3; p = 0.11) than in E carriers (adjusted OR = 2.18; CI: 0.75–6.29; Table 3, p = 0.15).

Although the number of African variants was too small to allow any stratified analysis, it is of relevance that 4 of the 8 Af-2 cases were carriers of DRB1*07-DQB1*02 haplotype (in the control group, this haplotype was identified in 47 of the 257 controls).

To verify if T to G substitution at 350 nucleotide position in the *E6 *viral oncogene could interfere in HLA association with ICC, we stratified cases according to this polymorphism and results of this analysis are on Table 4. The E6 83V group was composed of cases harbouring AA and the E-350G variants, while other E variants and Af variants formed the 83L group. Despite the observed inverse association between DQB1*05 and 83V cases (OR = 0.37; 95% CI: 0.15–0.89; Table 4, p = 0.03), the proportion of DQB1*05 carriers in 83L cases was not different from controls. Similar tendencies were also verified for the DQB1*0501 allele. We also found an inverse association between DRB1*04 and ICC cases positive for 83L E6 variants (adjusted OR = 0.27; 95% CI: 0.08–0.96; Table 4, p = 0.04), which was not observed for patients carrying 83V E6 variants.

**Table 4:** ORs and 95% CIs for HPV-16/E6-83V or E6-83L positive ICC according to HLA

	Controls	E6 83V Cases			E6 83L Cases		
			
HLA	C/N	C/N	Crude	Adjusted	C/N	Crude	Adjusted
							
	Controls	Cases	OR	OR (95% CI)	Cases	OR	OR (95% CI)
	n = 257	n = 40			n = 29		
DQA1							
*0101/04	79/178	7/33	0.48	0.46 (0.19–1.11)	10/19	1.19	1.13 (0.48–2.64)
*0102	80/177	10/30	0.74	0.74 (0.33–1.63)	14/15	2.07	2.24 (0.99–5.09)
DQB1							
*0301	78/179	11/29	0.87	0.88 (0.40–1.90)	11/18	1.40	1.29 (0.56–3.00)
*05	95/162	7/33	0.36	0.37 (0.15–0.89)	11/18	1.04	1.08 (0.47–2.49)
*0501	71/186	6/34	0.46	0.44 (0.17–1.14)	9/20	1.18	1.17 (0.48–2.81)
*06	87/170	16/24	1.30	1.29 (0.64–2.63)	14/15	1.82	1.93 (0.85–4.35)
*0602	44/213	9/31	1.41	1.31 (0.56–3.03)	10/19	2.55	2.33 (0.97–5.57)
DRB1							
*01	52/205	6/34	0.70	0.65 (0.25–1.09)	8/21	1.50	1.35 (0.54–3.39)
*0102	29/228	2/38	0.41	0.36 (0.08–1.66)	6/23	2.05	1.70 (0.60–4.82)
*04	74/183	12/28	1.06	1.11 (0.52–2.37)	3/26	0.29	0.27 (0.08–0.96)
*13	61/196	9/31	0.93	1.08 (0.47–2.47)	5/24	0.67	0.86(0.30–2.43)
*1302	27/230	0/40	0.00		3/26	0.98	1.32 (0.35–4.94)
*15	34/223	10/30	2.19	2.10 (0.91–4.86)	9/20	2.95	2.78 (1.11–6.92)
*1503	14/243	5/35	2.48	2.40 (0.77–7.51)	5/24	3.62	3.87 (1.20–12.52)
*DRB1-DQB1 haplotypes*							
0102-0501	29/228	2/38	0.41	0.36 (0.08–1.66)	6/23	2.05	1.70 (0.60–4.82)
15-0602	33/224	8/32	1.70	1.67 (0.68–4.07)	9/20	3.06	2.86 (1.14–7.16)
1503-0602	14/243	5/35	2.48	2.40 (0.77–7.51)	5/24	3.62	3.87 (1.20–12.52)
08041-0301	5/252	0/40			3/26	5.82	4.10 (0.68–24.81)
09012-0201/02	3/254	1/39	2.18	2.37 (0.22–25.12)	2/27	6.27	9.36 (1.38–63.25)

The frequency of DQA1*0102 carriers was higher in cases carrying 83L E6 variants (OR = 2.24; 95% CI: 0.99–5.09, p = 0.05) than in controls (Table 4). Increased risks were also seen for DRB1*15 alleles, particularly DRB1*1503. However, statistical significance was only unequivocal for cases with 83L variants (OR = 3.87, 95% CI: 1.20–12.52, p = 0.02). The analysis of haplotypes revealed a positive association between DRB1*15-DQB1*0602 and DRB1*1503-DQB1*0602 (OR = 2.86; 95% CI: 1.14–7.16, p = 0.02 and OR = 3.87; 95% CI: 1.20–12.52, p = 0.02 respectively; Table 4) with 83L carriers. Similar trends were found for 83V variants but they did not reach statistical significance. Despite the low frequency of the DRB1*09012-DQB1*0201/02 haplotype in this sample, it was positively associated with ICC carrying 83L variants (Table 4).

## Discussion

In this report we analyzed the interplay between HPV-16 variants and *HLA-DQA1, DQB1 *and *DRB1 *variability on the susceptibility to ICC in Brazilian women. We found similar frequencies of European (prototype) and AA HPV-16 variants in our group of cases and a lower proportion of African variants in samples from cervical cancer patients. In agreement with a previous study by our team of a different Brazilian population [6], the major European variant found in these samples was the prototype. Non-European variants are associated with greater risk of cervical lesions than European ones but results have not been uniform across populations. Some studies were conducted in European populations [20,27,37] and conflicting results were observed, probably due to the predominance of lower risk European variants in those populations [38]. The relatively high proportion of AA variants in our cases agrees with our previous observation in asymptomatic Brazilian women that indicate that these variants are associated with higher risks of HPV persistence and CIN development [6]. A case-control study done in Mexico also revealed a higher ICC risk among women carrying AA than E variants [7]. Data obtained from such admixtured populations in the Americas show a reproducible pattern of risk associated with non-European variants of HPV-16 [38]. The diverse distribution of HPV variants between cases and controls from several populations can reflect differences in their oncogenic potential. It has been suggested that differences in the LCR sequence might play a role in HPV-induced tumorigenesis, because nucleotide changes can alter promoter activity of the viral genome [39,40]. On the other hand, variations in coding regions, such as in E6 gene sequences could also explain this diverse oncogenic potential of variants through differential protein activity [8-11].

Several studies investigated the role of HLA class II genes and alleles in HPV related diseases. Positive associations with DRB1*15-DQB1*06 haplotype and inverse associations with HLA-DRB1*13 allele were described in different populations, including Brazilians [19], Americans/Hispanics [23] and Europeans [20,22]. In some of these studies, associations with cases harboring HPV-16 have been suggested: DRB1*0407-DQB1*0302 haplotype was positively associated with HPV-16 positive cases in Hispanic women [23]. Likewise, we have previously reported an association of DRB1*1503 with HPV positive ICC, with higher OR values when only HPV-16 cases were considered [19], but in this report we found similar results when compared women harboring E and AA variants. Since associations involving DR*15 alleles were observed in many populations [20,22,23,41-43], with different HPV variants, it is reasonable to speculate an effect of this allele on cervical disease independently of HPV-16 variants distribution.

On the other hand, stratification of cases according to HPV-16 variants allowed us to find that trends of inverse associations between HPV-16 cases with DQA1*0101/04 and DQB1*05 [19] are due to AA variants. Previous studies involving populations with few AA variants [26-28] did not report the association of AA cases with DQB1*05. Due to the high prevalence of E variants in these studies, they took into consideration mostly single nucleotide polymorphisms, as in the *E6 *gene, which generates variants designated L83V (T to G substitution at 350 nucleotide position). This substitution was associated with and increased risk for HPV persistence and ICC [12].

Inverse associations of cervical cancer with DRB1*13 group were found in Costa Rica [44] and Hispanic women from USA [23]; as well as Swedish [22]; French [45] and Dutch [42] populations. A trend for negative association was observed in American women [24], similar to what we observed previously between DRB1*1302 and ICC cases [19]. In our present study, the comparison of DRB1*1302 frequency between controls and European variants cases did not reveals any association, but, interestingly, this allele was not found in ICC cases positive for AA variants, suggesting a protective role of this allele to cases carrying AA variants.

We found a lower proportion of women carrying DR4 in 83L cases than in controls, and a similar trend was observed in the DRB1*04-DQB1*0302 haplotype comparison (data not shown). Interestingly, in a Swedish population, the frequency of DR*04-DQ*03 haplotype was higher in cases with 83V variant than in controls [28]; and similar trends were also described for Italian, Czech and other Swedish populations [26]. A study conducted in the British population revealed that HPV-16 E2 variants, which also present the E6 350G (83V), occurred more frequently in individuals with HLA-DRB1*0401-DQB1*0301 and DRB1*1101-DQB1*0301 haplotypes [46].

The influence of HPV-16 variants in HLA distribution in ICC cases suggests that variants can differ in their immunogenic potential. Although there are few studies and most of them restricted to small sample sizes, which limits the interpretation of some findings, it is possible that the associations can reflect alterations in the binding of viral epitopes to HLA molecules. The *DR4 *alleles found here are in linkage disequilibrium with *DQ3 *and it was already detected that a substitution of a residue in a HSV peptide impaired its binding to DQ3.2 molecule (allele DQB1*0302), but this altered peptide become able to bind to DQ3.1 and DQ3.3 molecules (DQB1*0301 and *0303 alleles, respectively) [47]. Functional consequences of alterations in viral proteins were demonstrated in animal models, and a single residue change in E6 protein of cottontail rabbit papillomavirus progressor strain led to high frequencies of spontaneous regressions in inbred rabbits [48]. Variations in E6 protein from AA and E variants differ in peptide positions (14H, 83V) that are encompassed by the epitopes described earlier [49,50], but further evaluation is warranted to elucidate a possible role of these substitutions in immune response.

In our previous report, we did not detect many differences when comparing HPV negative and positive controls [19]. However, HPV detection was performed in a single point in time. In our longitudinal study, different haplotypes were associated with HPV infections and/or persistence [51], but this study was conducted in a population of a different region in Brazil, which could reflect differences in HLA distribution. Bontkes et al. 1998 [27] suggest that immunogenetic factors associated with disease progression are different from those associated with susceptibility to HPV-16 infection. However, Beskow *et al*., 2002 [52] found a strong correlation between long-term infection and high viral load and between short-term infection and low viral load. They described that carriers of DRB1*1501-DQB1*0602 haplotype, had higher HPV-16 viral load than non-carriers [52] and that carriers of protective alleles (DRB1*1301 and DQB1*0603) have lower HPV-18/45 load compared to non carriers [53]. These results suggest an interaction between viral (as HPV types, variants and viral load) and host factors, and it is possible that HLA polymorphism may affect the immune reaction to the virus and indirectly play a role in the susceptibility to HPV-related lesions.

## Conclusion

Taken together our data suggest that HPV-16 variability influences the association between HLA polymorphism and ICC risk. Studies to elucidate its influence in immune responses to HPV, and its role in viral clearance or progression of HPV-related diseases are warranted.

## Abbreviations

CI: confidence intervals; HLA: human leukocyte antigen; HPV: human papillomavirus; OR: odds ratio; ICC: invasive cervical carcinoma; PCR: polymerase chain reaction; E: European variants of HPV-16; AA: Asian-American variants of HPV-16; Af-2: African-2 variants of HPV-16.

## Competing interests

The authors declare that they have no competing interests.

## Authors' contributions

PSAS carried out the HPV-16 variants typing, participated HLA typing and drafted the manuscript. PCM carried out the HLA typing and participated in study design. KBR performed the statistical analysis. MLP–E coordinated the HLA typing and participated in study design. ELF and LLV participated in the study design and supervised the study. All authors read and approved the final manuscript.

## Pre-publication history

The pre-publication history for this paper can be accessed here:


